# 2261: Incidence and predictors of noncompliance with evidence-based guidelines for early stage breast cancers using the National Cancer Data Base

**DOI:** 10.1017/cts.2017.256

**Published:** 2018-05-10

**Authors:** Albert Liao, Grant W. Carlson, John William Eley, Theresa W. Gillespie

## Abstract

OBJECTIVES/SPECIFIC AIMS: Evidence-based guideline-concordant care leads to better outcomes in patients with early stage breast cancer, including survival. However, previous studies of guideline compliance have been limited by small study sample sizes, localized geography, unknown causal factors, and lack of diverse population. We use a national database to assess socio-economic, clinical, and facility factors that impact treatment compliance with evidence-based guidelines from the American Society of Clinical Oncology (ASCO) and the National Comprehensive Cancer Network (NCCN). METHODS/STUDY POPULATION: This is a retrospective cohort study of the National Cancer Data Base Participant User File Breast 2014, which captures ~70%–80% of all newly diagnosed cancer cases in the United States. Female patients who were diagnosed with early stage breast adenocarcinoma (T0, T1, T1A, T1B, 2, 2A, or T2N1) from 2004 to 2014 were eligible for this study. RESULTS/ANTICIPATED RESULTS: A total of 807,314 patients were included in this study. Evidence-based guidelines examined with associated compliance rates include surgery completion (79.3% overall compliance), breast conserving surgery Versus mastectomy (88.05% vs. 11.95%, respectively), radiation after breast conserving surgery (77.5% overall compliance), HER2 testing (88.6% overall compliance), estrogen/progesterone receptor (ER/PR) testing (96.3% overall compliance), hormone treatment for positive ER/PR breast cancer (80.2% overall compliance), and sentinel lymph node biopsy completion (67.5% overall compliance). Univariate association between these guidelines and covariates such as facility type, facility location, age, race, insurance status, median income quartiles, achievement of high school degree, urban Versus rural, Charlson-Deyo score, year of diagnosis, and overall survival were assessed. Logistic regression analysis will be used to determine multivariate relationships between these characteristics and the probability that a patient will be compliant to guideline regimen. DISCUSSION/SIGNIFICANCE OF IMPACT: The results of this study will help identify socio-economic, clinical, and facility factors that influence guideline-concordant care and subsequent critical outcomes for patients with early stage breast cancer. Lack of guideline concordant care for specific stages of cancer or treatment modalities will point to a need for tailored interventions to enhance compliance. A prediction model will help identify the most important predictors of noncompliance in breast cancer treatment so noncompliance can be prevented in at-risk populations.

